# A systematic review of the cost-effectiveness of ultrasound in emergency care settings

**DOI:** 10.1186/s13089-021-00216-8

**Published:** 2021-03-09

**Authors:** Brian Lentz, Tiffany Fong, Randall Rhyne, Nicholas Risko

**Affiliations:** 1 Department of Emergency Medicine, Highland Hospital-Alameda Health System, 1411 E. 31st Street, QIC 22123, Oakland, CA 94602 USA; 2 Department of Emergency Medicine, Johns Hopkins University School of Medicine, 1800 Orleans St, Baltimore, MD 21287 USA

**Keywords:** Ultrasound, Emergency Medicine, Cost-effectiveness, Radiology, Health Economics

## Abstract

**Background:**

The use of ultrasound (US) in emergency departments (ED) has become widespread. This includes both traditional US scans performed by radiology departments as well as point-of-care US (POCUS) performed by bedside clinicians. There has been significant interest in better understanding the appropriate use of imaging and where opportunities to enhance cost-effectiveness may exist. The purpose of this systematic review is to identify published evidence surrounding the cost-effectiveness of US in the ED and to grade the quality of that evidence.

**Methods:**

We performed a systematic review of the literature following Preferred Reporting Items for Systematic Review and Meta-Analyses (PRISMA) guidelines. Studies were considered for inclusion if they were: (1) economic evaluations, (2) studied the clinical use of ultrasound, and (3) took place in an emergency care setting. Included studies were critically appraised using the Consolidated Health Economic Evaluation Reporting Standards checklist.

**Results:**

We identified 631 potentially relevant articles. Of these, 35 studies met all inclusion criteria and were eligible for data abstraction. In general, studies were supportive of the use of US. In particular, 11 studies formed a strong consensus that US enhanced cost-effectiveness in the investigation of pediatric appendicitis and 6 studies supported enhancements in the evaluation of abdominal trauma. Across the studies, weaknesses in methodology and reporting were common, such as lack of sensitivity analyses and inconsistent reporting of incremental cost-effectiveness ratios.

**Conclusions:**

The body of existing evidence, though limited, generally demonstrates that the inclusion of US in emergency care settings allows for more cost-effective care. The most definitive evidence for improvements in cost-effectiveness surround the evaluation of pediatric appendicitis, followed by the evaluation of abdominal trauma. POCUS outside of trauma has had mixed results.

## Background

Ultrasound (US) is well established as a safe and effective imaging modality for the rapid diagnosis and management of emergency conditions. At the bedside, it also improves success and patient safety during invasive procedures [1–5]. In the wake of technological advances and the increasing availability of imaging technologies, there has been considerable expansion of the use of clinical US—including both radiology-performed consultative studies and point-of-care ultrasound (POCUS) studies [6–11]. A number of initiatives have been promoted to encourage stewardship of imaging resources and the delivery of high-value care. These include the American College of Radiology’s “Appropriateness Criteria” which assists referring physicians in the selection of the most efficacious and medically-appropriate imaging [12]; the American Board of Internal Medicine Foundation’s “Choosing Wisely” initiative that advocates for use of modalities that are evidence-supported, non-duplicative, harm-free, and truly necessary [13]; and the American Institute of Ultrasound in Medicine’s “Ultrasound First” campaign that promotes the practice of ultrasound for its safety, effectiveness, and affordability [14].

There is a common assumption that US is cost-effective in emergency care settings as it is diagnostically valuable, rapid, and less expensive than other imaging modalities like computed tomography (CT) and magnetic resonance imaging (MRI) [15]. However, it is unclear whether the published data either support or refute this assertion.

Cost-effectiveness analysis is a tool that combines economic and health outcome data to produce standardized ratios of costs and benefits. The outputs of CEAs allow for comparison of diagnostics that vary by both price and clinical utility, and generate important data to support decisions related to health policy and investment. The validity and reliability of CEAs are highly dependent on the rigor with which they are conducted. In 2013, health economists developed consensus-based guidelines on the conduct and reporting of health economic evaluations, called the Consolidated Health Economic Evaluation Reporting Standards (CHEERS) checklist [16].

We present a systematic review of the published evidence surrounding the cost-effectiveness of US in emergency care settings. Our aim is to characterize the existing knowledge regarding the costs and benefits of emergency US, to examine the quality of cost-effectiveness studies using the CHEERS checklist, and to provide guidance for future research efforts.

## Methods

### Search strategy

**Fig. 1:**
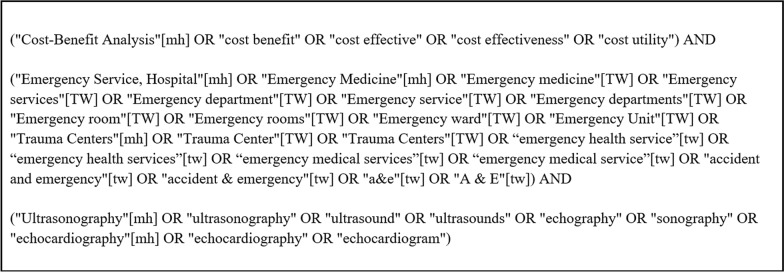
Search terms

A systematic review of the literature was performed using the Preferred Reporting Items for Systematic Review and Meta-Analyses (PRISMA) guidelines [17]. The review was conducted without date or language restrictions in five major databases (PubMed, Scopus, Embase, Web of Science, and Cochrane). The search terms used are found in Fig. 1.

### Selection of studies and data abstraction

All studies were collated and screened for eligibility through Covidence (www.covidence.org), an online platform for systematic review. Studies were considered for inclusion if they were: (1) economic evaluations that assessed costs and outcomes of comparative strategies; (2) studied the clinical use of US; and (3) took place in an emergency care setting. Two reviewers (BL, NR) with experience in reading, writing and reviewing economic evaluations independently assessed titles and abstracts for inclusion in a blinded fashion. Studies progressed to a full-text review if both reviewers agreed on the relevance based on the research question. Studies then underwent full text reviews for eligibility by each reviewer. Disagreements were resolved by consensus. The following information was extracted from each of the studies in the review: country, year of publication, intervention, comparator, time horizon (the time over which the costs and effect are measured), discount rate (the rate at which future costs and benefits are discounted), perspective (which costs are included or excluded), health outcome, sensitivity analyses (an assessment of the level of uncertainty in the modeling), and findings.

### Quality assessment

Two reviewers undertook critical appraisal of the included studies using the CHEERS checklist, which comprises 24 items determined by expert consensus to be of importance when reporting economic evaluations of health interventions [16]. The CHEERS checklist was developed to increase transparency and consistency in the reporting of economic evaluations of health interventions, and ultimately lead to better policy, program, and management decisions. A lower score means readers cannot fully understand the modeling assumptions and, therefore, must question the validity and reliability of the presented results. The checklist can also serve to highlight critical gaps in study design, for example when study authors have not described performing a sensitivity analysis to generate confidence intervals it is likely that this step was overlooked.

## Results

### Overview of included studies

**Fig. 2:**
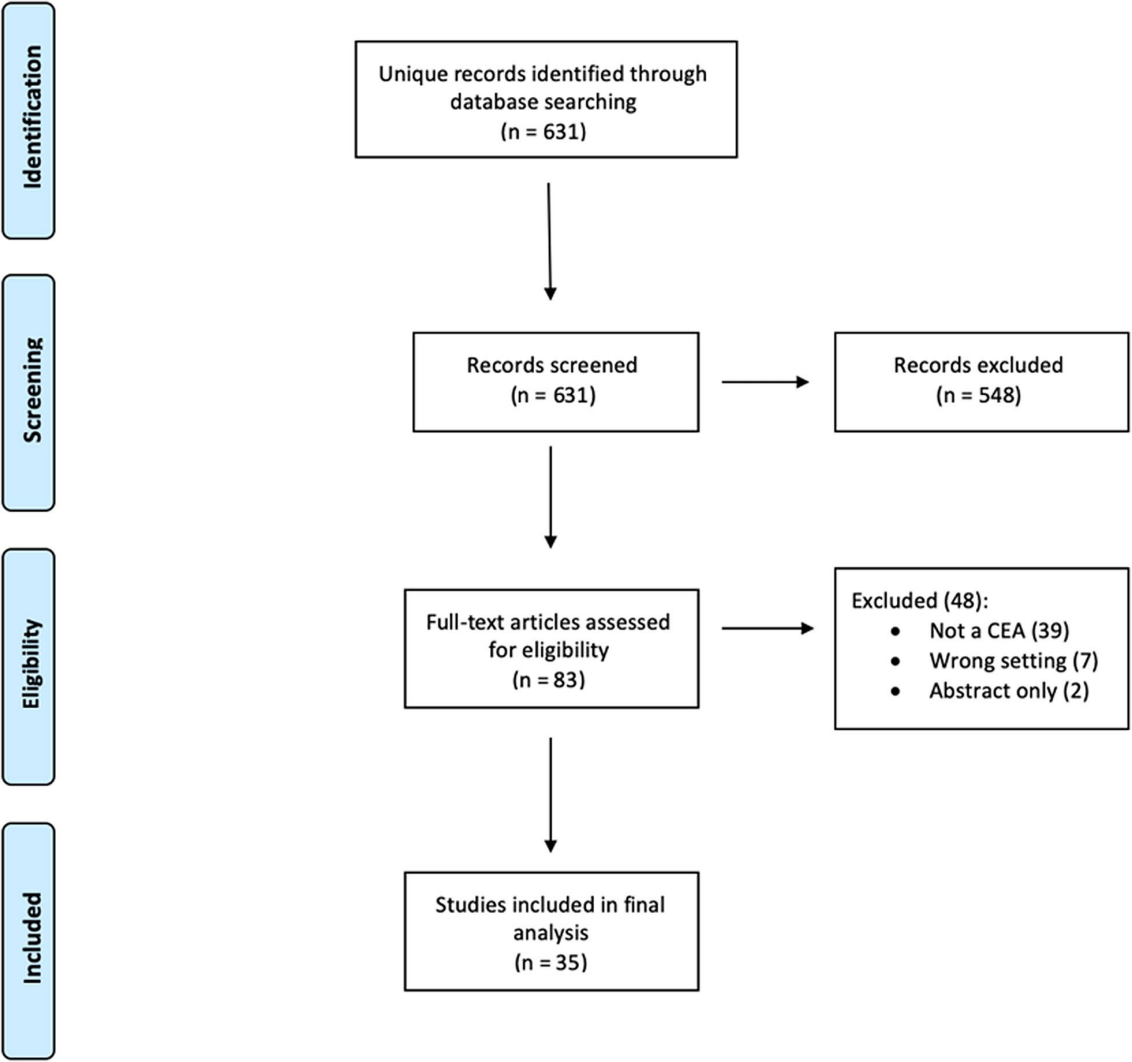
PRISMA flow diagram

The electronic searches identified 631 potentially relevant articles following the removal of duplicates. After screening titles and abstracts for eligibility, 83 articles remained for full text screening. Of these studies, 35 studies met all inclusion criteria and were eligible for data abstraction. Included studies ranged in publication date from 1996 to 2019, 20 were published in 2014 or later. Studies were excluded in the full-text phase for the following reasons: 39 were not economic evaluations or CEAs, seven were not relevant to the emergency care setting, and two were conference abstracts only. Details are further illustrated in our PRISMA Flow Diagram (Fig. 2).

### Description of included studies

The 35 included studies covered a broad range of applications within emergency US. The most comprehensive body of evidence surrounds the evaluation of pediatric appendicitis. This is followed by trauma, echocardiography, obstetric/gynecologic (OB/GYN), biliary, venous, and renal applications. A small group of studies also evaluate general US processes and impact in the emergency department (ED). Tables 1, 2, 3, 4, 5 and 6 present the principal findings of each study and our grade of the evidence using the CHEERS score.

**Table 1:** Evaluation of pediatric appendicitis

Study	Year	Findings	CHEERS Score (max 24)
Axelrod [18]	2000	US can be cost-saving as an adjunct to clinical decision making when deciding to observe or discharge benign pediatric abdominal pain; however, it is not cost-saving when appendicitis is strongly suspected	14
Pena [19]	2000	Staged imaging using US first followed by CT when US was negative or equivocal for pediatric appendicitis produced cost-savings compared to standard of care	11
Pershad [20]	2015	Using US and a clinical decision rule prior to CT for evaluation of pediatric appendicitis is more cost-effective than CT alone	17
Van Atta [21]	2015	Staged imaging with US first in pediatric patients with suspected appendicitis reduces overall cost through CT avoidance	10
Wagenaar [22]	2015	Staged imaging with US first in pediatric patients with suspected appendicitis reduces overall cost through CT avoidance and decreased length of stay	8
Gregory [23]	2016	The most cost-effective approach for assessing pediatric appendicitis is use of a clinical decision rule followed by staged imaging with US first	22
Anderson [24]	2017	A protocol using US and MRI for pediatric appendicitis successfully decreased use of CT scan though without any change in health outcomes and increased radiology costs over the study period	13
Imler [25]	2017	A comparison of US or MRI first in the evaluation of young patients for appendicitis showed higher overall costs and longer ED length of stay in the MRI group	10
Kharbanda [26]	2018	A study across nine pediatric EDs showed that in the evaluation of acute abdominal pain, US first sites had 5.2% lower total costs of treatment than CT first sites	17
Kobayashi [27]	2018	Implementation of an US first appendicitis pathway at a general hospital was unsuccessful in decreasing CT utilization due to poor adherence to the pathway	8
Nordin [28]	2018	Using a standardized reporting template to ensure US quality decreased equivocal studies and the need to perform CT scans for appendicitis in a pediatric ED, leading to further cost-savings estimated at nearly $150,000 per year	11

**Table 2:** Evaluation of trauma

Study	Year	Findings	CHEERS Score (max 24)
Branney [29]	1997	Ultrasound use to evaluate acute abdominal trauma can reduce costs through avoidance of peritoneal lavage and reduced length of stay	10
Partrick [30]	1998	Using US as a triage tool in pediatric blunt abdominal trauma may reduce costs through reduced CT scans	6
Arrillaga [31]	1999	In the evaluation of blunt abdominal trauma US decreases time to disposition and procedural costs	15
Frezza [32]	1999	Residents performing FAST is cost-saving compared to US technicians	8
Melniker [33]	2006	Utilizing FAST in the acute management of trauma patients reduces time to operating room, length of stay and overall costs	7
Hall [34]	2016	Performing the cardiac portion of FAST on blunt trauma patients is only cost-effective if they are hypotensive	23

**Table 3:** Evaluation of renal, biliary, and venous pathology

Study	Year	Findings	CHEERS Score (max 24)
Durston [35]	2001	Initial POCUS by ED physicians for right-upper quadrant pain followed by radiology department scans if needed, is the most cost-effective approach	12
Goodacre [36]	2006	Assessing for deep vein thrombosis with the Wells score and D-dimer prior to US improves cost-effectiveness by avoiding unnecessary scans	20
Young [37]	2010	There may be a large cost associated with repeating imaging tests for cholecystitis after they have already been performed by proficient ED physicians	9
Ward [38]	2010	In patients with high suspicion for pulmonary embolism but low likelihood of mortality, starting the diagnostic workup with US to assess for deep vein thrombosis and proceeding with only selective CT scanning is cost-effective	22
Melnikow [39]	2016	In patients with suspected kidney stones randomized to initial POCUS, radiology US, or CT there were no significant differences in either outcome or cost	14
Sternberg [40]	2017	An assessment of over 10,000 cases of acute renal colic showed that if US was ordered at the initial visit, overall imaging cost and radiation exposure were lower than if CT scan was ordered initially	9

**Table 4:** Evaluation of OB/GYN pathology

Study	Year	Findings	CHEERS score (max 24)
Hazlett [41]	1996	Transvaginal US is cost-saving compared to transabdominal as an initial test in the work-up of pelvic pain in the ED with the exception of patients in the second or third trimester of pregnancy with a palpable abdominal mass or with a full bladder	6
Durston [42]	2000	Initial POCUS by ED physicians in rule-out ectopic cases is cost-effective compared to initial radiology department imaging, although this may delay care in those with adnexal mass or tubal rupture	12
Al Wattar [43]	2014	Twenty-four hour US coverage for OB/GYN emergencies can avert admissions and cost-savings depends on volume	4

**Table 5:** Echocardiography

Study	Year	Findings	CHEERS Score (max 24)
Wyrick [44]	2008	Myocardial contrast echocardiography in the ED on selected chest pain patients may be cost-effective through the prevention of unnecessary admissions	16
Jasani [45]	2018	Patients with low-risk chest pain discharged after stress echocardiography in the ED have lower costs and similar health outcomes compared to similar patients admitted to the hospital	8
Baugh [46]	2019	Large variation exists in ordering practices for tests on older patients with syncope in the ED, with CT head and echocardiogram standing out as expensive tests with relatively low yield	11
Gc [47]	2019	In a modeled scenario based on pilot data, the use of tissue Doppler evaluation of diastolic dysfunction in patients with non-ST elevation acute coronary syndrome produced overall cost-savings by decreasing length of stay	21

**Table 6:** Miscellaneous studies

Study	Year	Findings	CHEERS Score (max 24)
McGahan [48]	2000	At a single institution, 24-h sonography coverage compared to on-call coverage generated net profit and may have improved patient outcomes	8
Morrow [49]	2015	Ballistics gel can be used to make an effective US training model at a cost-saving with respect to commercially available materials	7
Allen [50]	2017	Downstream imaging after an ED US is increased if the US is performed by a non-radiologist	12
Huang [51]	2017	Introducing a protocol for transcervical US for evaluating pediatric peritonsillar abscess decreased length of stay, surgical procedures and CT scans, however, did not decrease cost	12
Van Schaik [52]	2019	POCUS use in the ED likely produces cost savings by decreasing further diagnostic testing	12

### Evaluation of pediatric appendicitis

We identified 11 studies that assessed the cost-effectiveness of US in the evaluation of acute appendicitis [18–28]. The impetus to avoid radiation in the pediatric population has sparked substantial research interest in this area. Universally, these studies found that using US as the initial study to evaluate pediatric patients for appendicitis decreased radiation exposure, overall costs, and length of stay. These results held despite significant rates of inconclusive initial scans. The use of clinical decision rules and US quality templates further increased cost-effectiveness. One study did not find a significant change because of poor adherence to the US first protocol [27].

### Evaluation of trauma

Six studies examined US in the setting of acute trauma [29–34]. They found that US decreases time to the operating room for patients with injury, decreases the use of CT scans in children, decreases length of stay, and decreases invasive procedures such as peritoneal lavage in adults. One study specifically examined the cardiac portion of the focused assessment with sonography in trauma (FAST) exam in blunt trauma patients, finding it was only cost-effective in the setting of hypotension [34].

### Evaluation of renal, biliary, and venous pathology

Six studies examined US in the setting of renal, biliary, or venous pathology [35–40]. The use of US to evaluate acute flank pain had mixed findings. One study suggested no efficiency gains with US use due to high-numbers of subsequent CT orders, and another found that using US as the initial radiology modality decreases costs and radiation exposure despite a 20% subsequent use of CT to further evaluate patients [39, 40]. Two studies assessed the evaluation of biliary pathology. One study showed that in the evaluation of right-upper quadrant pain, initial POCUS by ED providers followed by radiology department scans if needed was the most cost-effective approach, and a second study described large costs associated with additional imaging after ED POCUS significant for acute cholecystitis [35, 37]. In the evaluation of venous thromboembolism, protocols using clinical screening tools, D-dimer testing, and US to decrease CT usage were the most cost-effective [36, 38].

### Evaluation of OB/GYN pathology

Three studies assessed US in the evaluation of emergency OB/GYN complaints [41–43]. One study showed that an initial approach of transvaginal instead of transabdominal US is cost-saving in all patients other than those in the second or third trimester of pregnancy [41]. Another study described benefits and drawbacks of POCUS as the initial test in a rule-out ectopic pregnancy scenario showing higher rates of detection of ectopic pregnancy at the first visit, but with an overall increase in number of US studies performed and the potential of other missed pathology [42]. Finally, a third study found that at high-volume sites 24 h a day US coverage to assess for OB/GYN pathology can avert admissions [43].

### Echocardiography

Four studies assessed echocardiography in the emergency care setting [44–47]. Three of the studies focused on decreasing hospitalization using US to provide earlier risk-stratification of patients with acute chest pain or acute cardiac issues [44, 45, 47]. One study assessed the cost-effectiveness of a variety of emergency department modalities to evaluate syncope in older patients, finding that CT head and echocardiography where among the most expensive and least revealing tests that are commonly ordered [46].

### Miscellaneous studies

Finally, we identified a collection of studies evaluating the downstream impact of emergency US on resource utilization [48–52]. One study found that patients who had POCUS as opposed to radiology scans were at increased risk of having further confirmatory studies ordered compared to those who had the initial US done by radiology [50]. Another small observational study of POCUS in the ED produced a contrary finding, noting that a bedside US performed by an emergency physician could lead to decreased testing afterwards [52].

### Quality assessment of included studies

While we tailored our search criteria to identify formal attempts at CEA, considerable variability remained in the quality of the economic evaluations conducted in the studies we identified. Most studies received about half the possible points on the CHEERS checklist. Inadequacy in the performance or the reporting of study perspectives, preference-based health outcomes (patient-reported preferences for different health states used to calculate metrics such as quality-adjusted life-years), modeling assumptions, transparency of costs, characterization of uncertainty, and incremental cost-effectiveness ratios contributed to the majority of downgrading. For example, only nine of the 35 studies presented any characterization of the uncertainty surrounding their cost-effectiveness results, only seven presented an incremental cost-effectiveness ratio, and only 11 explicitly stated the economic perspective from which they were calculating costs.

## Discussion

To our knowledge, this is the first systematic review to broadly evaluate the cost-effectiveness of US in the emergency care setting across all applications, including both radiology-performed and POCUS exams. The body of literature identified through this systematic review is fairly consistent in supporting the increased cost-effectiveness of integrating US in emergency care settings across a variety of applications. Healthcare systems have begun to focus on value-based approaches throughout the hospital including the ED [53], and the demonstration of cost-effectiveness of US has the potential to lead to an expansion of its role in ED patient care.

Of the studies identified in this systematic review, those pertaining to the cost-effectiveness of US in the ED evaluation for pediatric appendicitis were the most plentiful and consistent in demonstration of cost-effectiveness gains, particularly when used in conjunction with clinical decision rules [18–28]. Three of the studies identified in this review were amongst the most rigorous we identified using CHEERS methodology [20, 23, 26]. When combined with the decreased radiation exposure of this approach, these studies add to the case for the widespread adoption of an US first approach in the ED evaluation of pediatric appendicitis.

Use of POCUS evaluation of trauma is ubiquitous and recommended by the American College of Surgeons [54]. The six studies we identified demonstrated consistent cost-effectiveness gains when POCUS is used in the evaluation of trauma [29–34]. These studies, however, generally had poor cost-effectiveness methodology and reporting, with the exception of the investigation by Hall et al. which concluded that in blunt trauma patients the cardiac component of the FAST exam is only cost-effective when a patient is hypotensive [34].

Cost-effectiveness of consultative ED US was demonstrated across a variety of applications, including echocardiography, appendicitis, venous thromboembolism, renal, and obstetric. This is consistent with a prior narrative review that demonstrated increased cost-efficiency of US across the spectrum of clinical medicine [55].

The studies identified in this review generally support the cost-effectiveness of POCUS, although some reach the alternative conclusion. The aforementioned studies evaluating POCUS in trauma consistently demonstrated cost savings. Additionally, multiple studies demonstrated potential cost savings in POCUS for biliary and OB/GYN applications [35, 37, 42]. However, one study failed to identify cost savings in POCUS for patients with suspected kidney stones and another demonstrated additional downstream imaging after initial POCUS [39, 50]. Contrarily, a separate study identified in this review demonstrated that POCUS leads to decreased downstream testing and can lower direct and indirect costs associated with diagnostic workups [52]. Given the large number of supporting studies publication bias should be considered, but an assessment of this falls outside of the scope of our manuscript.

None of the studies included in this systematic review specifically address the widely-inferred cost-effectiveness of POCUS due to increased efficiency (US scans are both performed and interpreted at the bedside in real time by a clinician who can immediately integrate the results into patient management) and patient throughput, or the decreased adverse iatrogenic events due to US guidance of procedures [56–59]. This highlights a gap that could be addressed by future research.

This systematic review sought to identify all relevant articles by searching five databases without language or date restrictions; however, it remains possible that pertinent articles were missed. Multiple independent reviewers were utilized at each step of the study selection and data abstraction process to minimize the likelihood of excluding relevant studies. Additionally, the mixed quality of methodology and reporting surrounding the economic evaluations conducted in these studies is notable. Most authors did not frame their analysis as a formal cost-effectiveness study, which may have led to the lack of robust methodology. Both past and recent reviews of cost-effectiveness studies in radiology have highlighted the need for standardized approaches [60, 61]. This limited utilization of established health economics modeling and reporting standards calls into question the reliability of results and makes comparability across studies difficult.

## Conclusions

In summary, the body of existing evidence, though limited, is generally supportive of the cost-effectiveness of US in the emergency care setting. The strongest evidence of cost-effectiveness of ultrasound in the ED exists in the evaluation of pediatric appendicitis and of abdominal trauma. Further study, using established best-practice standards in the methodology of cost-effectiveness analysis, is needed to definitively establish the cost-effectiveness of consultative and POCUS across a variety of applications in emergency care settings.

## Data Availability

All data generated or analyzed during this study are included in this published article.

## Abbreviations

CEA: Cost-effectiveness analysis
ED: Emergency Department
FAST: Focused Assessment with Sonography in Trauma
POCUS: Point-of-Care Ultrasound
US: Ultrasound
CT: Computed Tomography
PRISMA: Preferred Reporting Items for Systematic Review and Meta-Analyses
CHEERS: Consolidated Health Economic Evaluation Reporting Standards
OB/GYN: Obstetric/Gynecologic
MRI: Magnetic Resonance Imaging
